# Perovskite Light-Emitting Devices with Doped Hole Transporting Layer

**DOI:** 10.3390/molecules26061670

**Published:** 2021-03-17

**Authors:** Zhiwei Peng, Yuhan Gao, Guohua Xie

**Affiliations:** 1 Sauvage Center for Molecular Sciences, Hubei Key Lab on Organic and Polymeric Optoelectronic Materials, Department of Chemistry, Wuhan University, Wuhan 430072, China; leojpeng@whu.edu.cn (Z.P.); 2015301750036@whu.edu.cn (Y.G.); 2 Wuhan National Laboratory for Optoelectronics, Huazhong University of Science and Technology, Wuhan 430074, China; 3 Guangdong Provincial Key Laboratory of Luminescence from Molecular Aggregates, South China University of Technology, Guangzhou 510641, China

**Keywords:** perovskite quantum dots, light-emitting diodes, solution process, hole transporting layer

## Abstract

Perovskite quantum dots (PQDs) have drawn global attention in recent years and have been used in a range of semiconductor devices, especially for light-emitting diodes (LEDs). However, because of the nature of low-conductive ligands of PQDs and surface and bulk defects in the devices, charge injection and transport should be carefully managed in order to maximize the electroluminescent performances. In this study, we employed three p-dopants, i.e., 2,3,5,6-tetrafluoro-7,7,8,8-tetracyanoquinodimethane (F4-TCNQ), 1,3,4,5,7,8-hexafluoro-11,11,12,12-tetracyanonaphtho-2,6-quinodimethane (F6-TCNNQ), and 11,11,12,12-tetracyanonaphtho-2,6-quinodimethane (TCNH14), respectively doped into the commonly used hole transporting layer (HTL) poly[bis(4-phenyl)(2,4,6-trimethylphenyl)amine] (PTAA). Compared with the devices with the neat PTAA, those with the doped PTAA as the HTLs achieved the improved electroluminescent performances. In particular, the device with the strong oxidant F4-TCNQ exhibited an improvement factor of 27% in the peak external quantum efficiency compared with the control device with the neat PTAA. The capacitance and transient electroluminescent measurements were carried out to identify the imperceptible interactions in the doped HTL and at the interface between the HTL and PQDs.

## 1. Introduction

In recent years, perovskite quantum dots (PQDs) have drawn great attention due to their promising applications in semiconductor devices, including photovoltaics [1,2,3], lasers [4,5], and light-emitting diodes (LEDs) [2,6,7,8]. As a new type of quantum dots, PQDs have exhibited excellent performances owing to their unique optoelectronic properties depending on size and composition [9], including narrow bandwidth, tunable colors, and high photoluminescence quantum yield (PLQY) [7,10,11]. However, the poorly conductive ligands hinder the charge injection and thus the overall electroluminescent (EL) performances [12]. Due to the surface and bulk defects, surface traps and ion migration may be induced, which worsens charge injection and transport properties [13]. Furthermore, the optical properties of PQDs are easily influenced by their surrounding environment, which leads to the relatively low stability of PQDs under EL process [14]. Therefore, it is of fundamental importance to improve the charge injection and transport in the LEDs based on PQDs.

Electrical doping (p- and n-type) has been proved to be an efficient way to enhance the device performances. The admixing of the electrical dopant into the matrix of an organic semiconductor could lower the injection barrier and simultaneously increase the density of charge carriers, which result in the enhanced electric conductivity [15,16]. P-type dopants are applied in hole transporting layers (HTLs), which typically have deep lowest unoccupied molecular orbitals (LUMOs), i.e., large electron affinities (EAs). One class of the most widely used p-dopants has quinone structures with the unsatisfied aromatic rings, among which the prototypical compound is 7,7,8,8-tetracyano-1,4-quinodimethane (TCNQ) [17]. TCNQ was extensively studied because of its ability to form charge transfer salts with the extraordinarily high conductivities [18]. Due to the relatively smaller EA of TCNQ (4.3 eV) [19], a variety of TCNQ-derivatives were synthesized to match the highest occupied molecular orbitals (HOMOs) of the HTLs, e.g., 2,3,5,6-tetrafluoro-7,7,8,8-tetracyanoquinodimethane (F4-TCNQ), 11,11,12,12-tetracyanonaphtho-2,6-quinodimethane (TCNH14), and 1,3,4,5,7,8-hexafluoro-11,11,12,12-tetracyanonaphtho-2,6-quinodimethane (F6-TCNNQ). In recent years, Yan et al. proved that 0.01% doping of F4-TCNQ in polymer solar cells could dramatically decrease trap density and increase carrier mobility [20]. Mun et al. used F4-TCNQ as an additive to fabricate stretchable transistors with high mobility and stability [21]. Karpov et al. doped F6-TCNNQ into a donor–accepter copolymer and achieved a conductivity of 2 S/m. They also found that F6-TCNNQ-doped poly(3-hexylthiophene) (P3HT) exhibited a conductivity up to 7 S/m, which was higher than that of the P3HT:F4-TCNQ system [22]. Chen et al. applied F6-TCNNQ-doped nickel oxide as the HTL in perovskite solar cells, which decreased the charge extraction barrier between the HTL and the active layer and finally led to a high power conversion efficiency around 20% [23]. Beyer et al. doped F6-TCNNQ and TCNH-14 into a planar organic semiconductor DBTTF and found that both dopants could increase the conductivity, although the latter had a relatively smaller EA [24]. In 2019, Kiefer et al. even found the double doping phenomenon may generically exist in strong dopants such as F4-TCNQ, which led to the exceptionally high doping efficiency [25]. Therefore, it is conceivable such oxidant dopants could improve charge injection and transport of the PQD-based LEDs and thus the overall EL performances.

In this investigation, the three dopants mentioned above were respectively added into poly[bis(4-phenyl)(2,4,6-trimethylphenyl)amine] (PTAA), which is a typical HTL for perovskite-based devices [26,27,28]. The doping effects and the influence on the device performances were systematically studied. As a result, the F4-TCNQ-doped device achieved a maximum external quantum efficiency (EQE) of 5.6%, which was 27% higher than that of the device with the pristine PTAA as the HTL.

## 2. Results

### 2.1. Photophysical Properties

**Figure 1:**
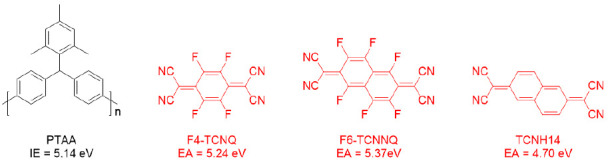
Chemical structures of the compounds used in the doped hole transporting layer (HTL). Ionization energy (IE) of poly[bis(4-phenyl)(2,4,6-trimethylphenyl)amine] (PTAA) and the electron affinity (EA) of each dopant, i.e., 2,3,5,6-tetrafluoro-7,7,8,8-tetracyanoquinodimethane (F4-TCNQ), 1,3,4,5,7,8-hexafluoro-11,11,12,12-tetracyanonaphtho-2,6-quinodimethane (F6-TCNNQ), and 11,11,12,12-tetracyanonaphtho-2,6-quinodimethane (TCNH14), were indicated correspondingly.

**Figure 2:**
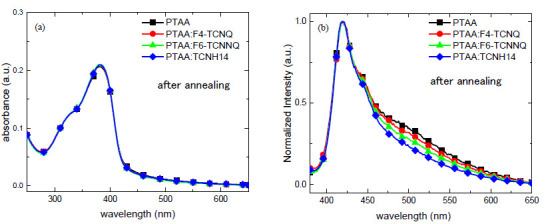
(**a**) UV-Vis absorbance and (**b**) normalized photoluminescence emission of the doped HTL layers with a fixed doping ratio of 96:4 by weight.

The chemical structures of PTAA and the dopants are shown in Figure 1, where the ionization energy (IE) of PTAA [29] and the EAs of dopants [23,24,30] are noted. Unlike TCNH14, F4-TCNQ and F6-TCNQ exhibited the more suitable LUMOs in the proximity of the HOMO level of PTAA, which may allow more efficient charge transfer when mixed together. To unravel the interaction between the dopants and the HTL PTAA, the absorbance and emission spectra of the four different PTAA films spin-coated on the quartz substrates were measured. As shown in Figure 2a, neither obvious charge transfer complexes (CTCs) nor ion absorption could be found in any doped HTL in the visible range, even though F4-TCNQ and F6-TCNNQ had the well-matched EAs with the IE of PTAA. This may be attributed to the weak and imperceptible charge transfer or the highly absorptive polymer PTAA.

However, as shown in Figure 2b, the inhibitory effects of all the dopants on the fluorescence of PTAA were detectable in the range of 450–650 nm, which was presumably assigned to the emission of aggregated triphenylamine units. This means that the dopant molecules interact with PTAA. In the presence of an oxidant dopant in the HTL, the fluorescence quenching of the polymer segment of PTAA might be one of the indicators of charge transfer, which could lead to more free charge carriers and thus better conductivity.

### 2.2. Device Performances

**Figure 3:**
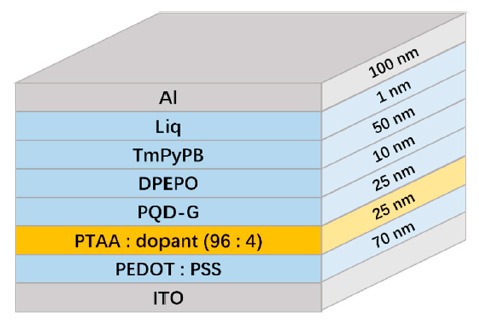
Device structure of the multi-layer stacks used in this investigation.

To validate the functionality of the doped PTAA as HTLs, four different devices were fabricated. Device A denotes the neat PTAA as the HTL. Meanwhile, devices B, C, and D, respectively, consisted of the doped PTAA with F4-TCNQ, F6-TCNNQ, and TCNH14. The architecture of the devices is shown in Figure 3. Here, PQD-G represents the green color PQDs, PEDOT:PSS refers to poly(styrenesulfonate)-doped poly(3,4-ethylenedioxythioophene) as the hole injection layer (HIL), DPEPO bis[2-((oxo)diphenylphosphino)phenyl] ether as the hole blocking layer, TmPyPB 1,3,5-tri[(3-pyridyl)-phen-3-yl]benzene as the electron transporting layer, and Liq 8-hydroxyquinolinolato-lithium as the electron injecting layer, respectively.

**Figure 4:**
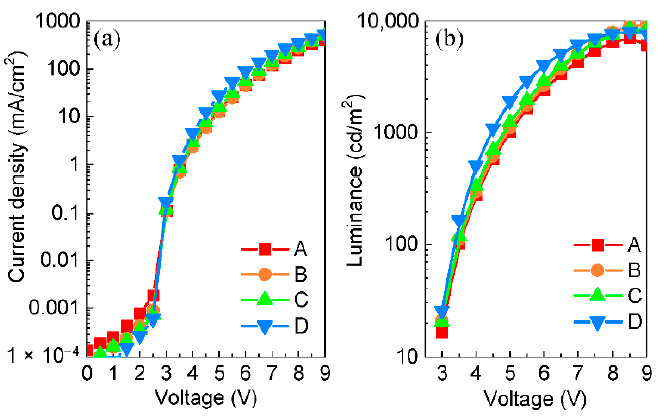
(**a**) Current density–voltage and (**b**) luminance–voltage curves of the devices.

As shown in Figure 4a, at a current density of 10 mA/cm^2^, device B with the F4-TCNQ-doped HTL exhibited the slightly increased driving voltage from about 4.80 V to 4.85 V, compared with the reference device A with the neat PTAA as the HTL. In contrast, the driving voltages of devices B and C corresponding to F6-TCNNQ and TCNH14 used as the dopants were only 4.70 V and 4.38 V, respectively, at the same current density mentioned above. It is indicative that the devices with the doped HTL display small ohmic loss. It is worth mentioning that device D with TCNH14 was most conductive when the driving voltage was above 3 V. More specifically, device D had a current density of 27.9 mA/cm^2^ under 5.0 V, which was a factor of 2 higher than that of device A with only 13.5 mA/cm^2^. At the same driving voltage, devices B and C had current densities of 12.7 and 16.0 mA/cm^2^, respectively.

Figure 4b shows the luminance–voltage relationship of the devices. Due to the inferior conductivity, device A reached the luminance of 1000 cd/m^2^ at 5 V, while less than 4.9 V was required for devices B and C. Noticeably, device D with PTAA:TCNH14 as the doped HTL only required 4.4 V to achieve the same luminance due to the superior charge carrier injection and transport. The maximum brightness of the doped devices was more than 8000 cd/m^2^ while that of the non-doped one was only 7105 cd/m^2^. These results manifest that all three dopants could positively influence the charge transport of the doped HTLs, which dominates the conductivity.

**Figure 5:**
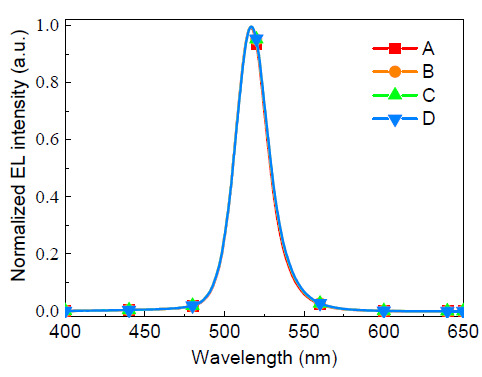
Normalized EL spectra of the devices.

Figure 5 illustrates the normalized EL spectra of the four devices. All the devices had a same peak at 516 nm, accompanied with the small full-width at half-maximum (FWHM) of about 25 nm. The identical EL profiles of the devices indicate that all the radiative excitons originate from the green PQDs, although the HTLs are emissive as shown in Figure 2b.

**Figure 6:**
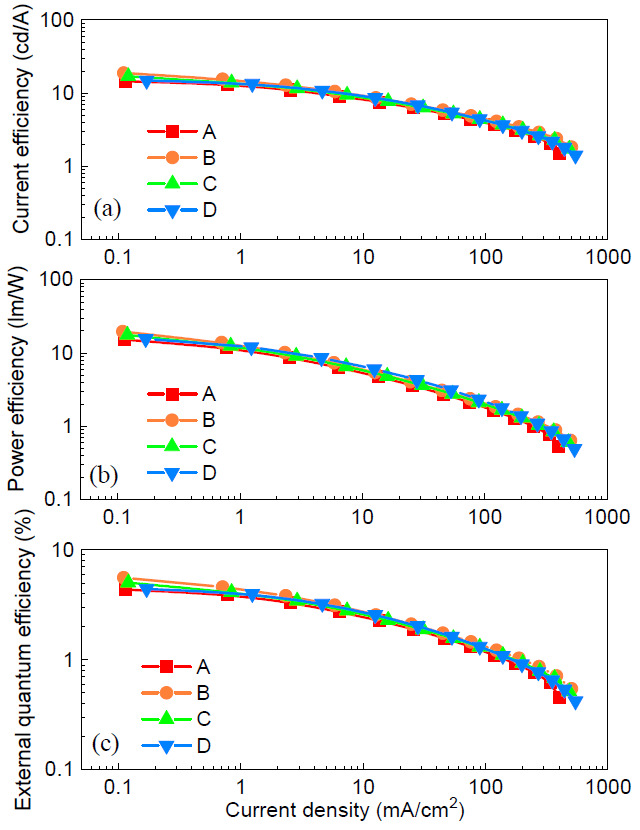
**(a**) Current efficiency, (**b**) power efficiency, and (**c**) external quantum efficiency (EQE) curves of the devices.

The EL efficiencies of the devices are shown in Figure 6. Device A with the neat PTAA had a maximum current efficiency of 15.6 cd/A (see Figure 6a). In contrast, device B with the dopant F4-TCNQ achieved the highest value of 18.8 cd/A among the four devices, which corresponds to an improvement factor of 21%, compared with the control device A. The maximum current efficiency of device C with F6-TCNNQ was 17.0 cd/A. However, the peak value of device D with TCNH14 was down to 14.9 cd/A despite that the higher current density and brightness were concurrently maintained at a forward driving voltage, shown in Figure 4a,b. This is ascribed to the imbalanced charge carriers in the emissive zone. As shown in Figure 6b, the maximum power efficiency of device B was up to 19.7 lm/W, i.e., 29% higher than that of device A (15.3 lm/W). Devices C and D obtained the maximum power efficiencies of 17.7 and 15.6 lm/W, respectively. The peak EQEs of devices A, B, and C with the doped HTL were 5.6%, 5.0%, and 4.4% (see Figure 6c), respectively, while that of device A was 4.4%. It is now convincing that the doped HTLs play an important role in ruling the charge carrier injection and transport and thus the charge balance in the emissive PQD layer.

## 3. Discussion

**Figure 7:**
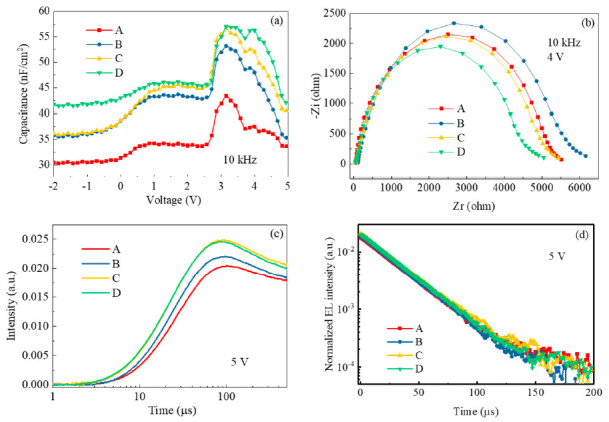
(**a**) Capacitance–voltage curves, (**b**) Nyquist plot recorded at 10 kHz, (**c**) rising time, and (**d**) transient EL after pulse off at 5 V of the devices.

To further clarify the doping effects of the HTLs, the impedance measurement was conducted on the four devices. Figure 7a shows the changes of the capacitance at different driving voltages. The capacitances at 10 kHz of the three devices with the doped HTLs are significantly higher than that of the non-doped one, showing that doping increases the density of free charge in the HTL and thus improves the conductivity. Device D had the largest capacitance, which is consistent with the largest current density shown in Figure 4a. The Nyquist plots recorded at 4 V (see Figure 7b) display similar trends shown in Figure 7a, except device D seems more resistive that device A without the dopant in PTAA at 10 kHz. This may be attributed to the smallest molecular structure and thus unstable morphology/electrical property after continuous driving, which generates joule heat. Figure 7c represents the rising time of the device after applying a pulsed voltage of 5 V. It is clear that the devices with the doped HTL were switched on more quickly than device A with the neat PTAA. The more charge carriers generated, the faster response to the pulse. This is very beneficial for achieving high refresh rates in the high-end active matrix displays. Nevertheless, the transient EL curves (see Figure 7d) are almost the same after turning off the pulsed voltage. The long delayed EL times are closely related to the architecture, which is challenging to resolve in the multilayer stacks with the different sources of traps and defects. In three doped devices, the imperceptible charge transfer between the dopants and the HTL indeed shapes the steady-state and transient EL properties of the devices with the novel PQDs. With the suitable EAs of the dopants, it is confirmed in this investigation that vital charge transfer interactions exist between the HTL and fluorinated and non-fluorinated dopants [24].

## 4. Materials and Methods

All materials were obtained and used without further purification. The PQDs were synthesized according to the literature [31]. PEDOT:PSS and PTAA were purchased from Xi’an Polymer Light Technology Corporation. TCNH14 was purchased from Changchun Tuo Cai Technology Co., Ltd. The other materials used in this study were ordered from Luminescence Technology Corporation.

The indium-tin-oxide (ITO) substrates were cleaned with acetone and ethanol consecutively in an ultrasonic bath for 10 min. After treating in the UV-ozone chamber (SenLights Corporation, Osaka, Japan) for 20 min, the substrates were spin-coated (KW-4A CAS, Beijing, China) with a layer of PEDOT:PSS, which was annealed at 120 °C for 10 min. Then the samples were loaded in the N_2_-filled glovebox (MBraun, München, Germany). The doped hole transporting layer was prepared by mixing the dopant (2 mg/mL) and PTAA (5 mg/mL) in chlorobenzene solution to meet the weight ratio of 96:4 and then spin-coated at 1000 rpm for 30 s onto the hole injecting layer PEDOT:PSS. After baking the hole transporting layer at 100 °C for 10 min on a hot plate, the emitting layer was spin-coated at 1000 rpm for 30 s and then annealed at 50 °C for 10 min before being loaded into the high-vacuum chamber (MB-200MOD MBraun, München, Germany). Then the hole blocking layer DPEPO, the electron transporting layer TmPyPB, the electron injection layer Liq, and the cathode Al were sequentially deposited in a high-vacuum chamber. Finally, all the samples were encapsulated with the UV curable glue before taken out from the glovebox.

All the measurements were operated at room temperature. UV-vis absorption spectra were recorded on a Shimadzu UV-2700 (Shimadzu, Scientific Instruments, Kyoto, Japan) recording spectrophotometer. Photoluminescence spectra were recorded on a Hitachi F-4600 (Hitachi, Tokyo, Japan) fluorescence spectrophotometer. The electroluminescence properties were measured with a Photo research SpectraScan PR735 spectrometer (Photo Research Inc., Chatsworth, CA, USA) and a Keithley 2400 source meter unit (Keithley Instruments, Cleveland, OH, USA). The impedance and transient EL properties were measured with Paios 4.0 (Fluxim, Winterthru, Switzerland).

## 5. Conclusions

Three dopants of the TCNQ derivatives were employed in the commonly used HTL for the LEDs based on the perovskite quantum dots as the emissive layer, resulting in the improved device performances. Different from other p-doped systems, no direct/obvious charge transfer could be found in these doped PTAA layers based on the absorption. However, the luminescence quenching of PTAA segments, the steady-state and transient EL, and the impedance measurements consistently evidence the interactions between the dopants and the HTL and the influence on the EL performances. Consequently, the luminance was improved from 7105 cd/m^2^ of the non-doped device to 9257 cd/m^2^ of the device with F4-TCNQ. Simultaneously, the external quantum efficiency was elevated from 4.4% to 5.6% by addressing the tradeoff between the charge carrier injection/transport in the HTL and balance in the emissive zone. This finding suggests that electrical doping is feasible to improve the electroluminescent performances of the solution-processed LEDs. By aligning the energy levels of the dopants with the hole transporting layer, the charge injection barrier could be lower and thus reduce the ohmic loss, which provides a universal strategy to maximize the performances of solution-processed lighting-emitting devices, photovoltaic devices, and transistors.

## Data Availability

The data presented in this study is available in article or from the corresponding author.

## Sample Availability

Samples of the compounds are available from the authors based on agreement.
